# Pro-vegetarian food patterns and cardiometabolic risk in the PREDIMED-Plus study: a cross-sectional baseline analysis

**DOI:** 10.1007/s00394-021-02647-4

**Published:** 2021-08-09

**Authors:** Alejandro Oncina-Cánovas, Jesús Vioque, Sandra González-Palacios, Miguel Ángel Martínez-González, Jordi Salas-Salvadó, Dolores Corella, Dolores Zomeño, J. Alfredo Martínez, Ángel M. Alonso-Gómez, Julia Wärnberg, Dora Romaguera, José López-Miranda, Ramon Estruch, Rosa M. Bernal-Lopez, José Lapetra, J. Luís Serra-Majem, Aurora Bueno-Cavanillas, Josep A. Tur, Vicente Martín-Sánchez, Xavier Pintó, Miguel Delgado-Rodríguez, Pilar Matía-Martín, Josep Vidal, Clotilde Vázquez, Lidia Daimiel, Emili Ros, Estefanía Toledo, Nancy Babio, Jose V. Sorli, Helmut Schröder, María Angeles Zulet, Carolina Sorto-Sánchez, Francisco Javier Barón-López, Laura Compañ-Gabucio, Marga Morey, Antonio García-Ríos, Rosa Casas, Ana María Gómez-Pérez, José Manuel Santos-Lozano, Zenaida Vázquez-Ruiz, Stephanie K. Nishi, Eva M. Asensio, Núria Soldevila, Itziar Abete, Leire Goicolea-Güemez, Pilar Buil-Cosiales, Jesús F. García-Gavilán, Erik Canals, Laura Torres-Collado, Manuela García-de-la-Hera

**Affiliations:** 1 CIBER de Epidemiología y Salud Pública (CIBERESP), Instituto de Salud Carlos III, Madrid, Spain; 2 Instituto de Investigación Sanitaria y Biomédica de Alicante, Universidad Miguel Hernández (ISABIAL-UMH), Alicante, Spain; 3 Nutritional Epidemiology Unit, University Miguel Hernandez, Alicante, Spain; 4 Centro de Investigación Biomédica en Red Fisiopatología de La Obesidad y La Nutrición (CIBEROBN), Institute of Health Carlos III, Madrid, Spain; 5 Department of Preventive Medicine and Public Health, IDISNA, University of Navarra, Pamplona, Spain; 6 Department of Nutrition, Harvard T.H. Chan School of Public Health, Boston, MA USA; 7 Departament de Bioquímica I Biotecnologia, Universitat Rovira I Virgili, Unitat de Nutrició, Reus, Spain; 8 Nutrition Unit, University Hospital of Sant Joan de Reus, Reus, Spain; 9 Institut D’Investigació Sanitària Pere Virgili (IISPV), Reus, Spain; 10 Department of Preventive Medicine, University of Valencia, Valencia, Spain; 11 Unit of Cardiovascular Risk and Nutrition, Institut Hospital del Mar de Investigaciones Médicas Municipal D’Investigació Médica (IMIM), Barcelona, Spain; 12 Department of Nutrition, Food Sciences, and Physiology, University of Navarra, Pamplona, Spain; 13 Precision Nutrition Program, IMDEA Food, CEI UAM + CSIC, Madrid, Spain; 14 Bioaraba Health Research Institute, Cardiovascular, Respiratory and Metabolic Area, Osakidetza Basque Health Service, Araba University Hospital, University of the Basque Country UPV/EHU, Vitoria-Gasteiz, Spain; 15 EpiPHAAN Research Group, School of Health Sciences, Instituto de Investigación Biomédica de Málaga (IBIMA), University of Málaga, 29010 Málaga, Spain; 16 Health Research Institute of the Balearic Islands (IdISBa), Palma de Mallorca, Spain; 17 Department of Internal Medicine, Maimonides Biomedical Research Institute of Cordoba (IMIBIC), Reina Sofia University Hospital, University of Cordoba, Cordoba, Spain; 18 Department of Internal Medicine, Institut D’Investigacions Biomèdiques August Pi Sunyer (IDIBAPS), Hospital Clinic, University of Barcelona, Barcelona, Spain; 19 Department of Endocrinology, Instituto de Investigación Biomédica de Málaga (IBIMA), Virgen de La Victoria Hospital, University of Málaga, Málaga, Spain; 20 Department of Family Medicine, Research Unit, Distrito Sanitario Atención Primaria Sevilla, Sevilla, Spain; 21 Research Institute of Biomedical and Health Sciences (IUIBS), University of Las Palmas de Gran Canaria and Centro Hospitalario Universitario Insular Materno Infantil (CHUIMI), Canarian Health Service, Las Palmas de Gran Canaria, Spain; 22 Department of Preventive Medicine and Public Health, University of Granada, Granada, Spain; 23 Research Group on Community Nutrition and Oxidative Stress, University of Balearic Islands, Palma de Mallorca, Spain; 24 Institute of Biomedicine (IBIOMED), University of León, León, Spain; 25 Lipids and Vascular Risk Unit, Internal Medicine, Hospital Universitario de Bellvitge, Hospitalet de Llobregat, Barcelona, Spain; 26 Division of Preventive Medicine, Faculty of Medicine, University of Jaén, Jaén, Spain; 27 Department of Endocrinology and Nutrition, Instituto de Investigación Sanitaria Hospital Clínico San Carlos (IdISSC), Madrid, Spain; 28 CIBER Diabetes y Enfermedades Metabólicas (CIBERDEM), Instituto de Salud Carlos III (ISCIII), Madrid, Spain; 29 Department of Endocrinology, Institut D’Investigacions Biomédiques August Pi Sunyer (IDIBAPS), Hospital Clinic, University of Barcelona, Barcelona, Spain; 30 Department of Endocrinology and Nutrition, Hospital Fundación Jimenez Díaz, Instituto de Investigaciones Biomédicas IISFJD, University Autonoma, Madrid, Spain; 31 Nutritional Control of the Epigenome Group, IMDEA Food, CEI UAM + CSIC, Madrid, Spain; 32 Lipid Clinic, Department of Endocrinology and Nutrition, Institut D’Investigacions Biomèdiques August Pi Sunyer (IDIBAPS), Hospital Clínic, Barcelona, Spain; 33 Dpto. Salud Pública, Facultad de Medicina, Hª de La Ciencia y Ginecología, Avda. Ramón y Cajal s/n, Sant Joan d’Alacant, 03550 Alicante, Spain

**Keywords:** Dietary food patterns, Cardiometabolic risk, Metabolic syndrome, Pro-vegetarian

## Abstract

**Purpose:**

We explored the cross-sectional association between the adherence to three different provegetarian (PVG) food patterns defined as general (gPVG), healthful (hPVG) and unhealthful (uPVG), and the cardiometabolic risk in adults with metabolic syndrome (MetS) of the PREDIMED-Plus randomized intervention study.

**Methods:**

We performed a cross-sectional analysis of baseline data from 6439 participants of the PREDIMED-Plus randomized intervention study. The gPVG food pattern was built by positively scoring plant foods (vegetables/fruits/legumes/grains/potatoes/nuts/olive oil) and negatively scoring, animal foods (meat and meat products/animal fats/eggs/fish and seafood/dairy products). The hPVG and uPVG were generated from the gPVG by adding four new food groups (tea and coffee/fruit juices/sugar-sweetened beverages/sweets and desserts), splitting grains and potatoes and scoring them differently. Multivariable-adjusted robust linear regression using MM-type estimator was used to assess the association between PVG food patterns and the standardized Metabolic Syndrome score (MetS z-score), a composed index that has been previously used to ascertain the cardiometabolic risk, adjusting for potential confounders.

**Results:**

A higher adherence to the gPVG and hPVG was associated with lower cardiometabolic risk in multivariable models. The regression coefficients for 5th vs. 1st quintile were − 0.16 (95% CI: − 0.33 to 0.01) for gPVG (*p* trend: 0.015), and − 0.23 (95% CI: − 0.41 to − 0.05) for hPVG (*p* trend: 0.016). In contrast, a higher adherence to the uPVG was associated with higher cardiometabolic risk, 0.21 (95% CI: 0.04 to 0.38) (*p* trend: 0.019).

**Conclusion:**

Higher adherence to gPVG and hPVG food patterns was generally associated with lower cardiovascular risk, whereas higher adherence to uPVG was associated to higher cardiovascular risk.

## Introduction

Cardiovascular disease (CVD) is the leading cause of premature death and chronic disability worldwide and increases the costs of the healthcare system [1]. Therefore, it is urgent and a priority to provide solutions based on the best scientific evidence for early detection and prevention [2]. Cardiometabolic risk indices or equations are a useful tool to early evaluate CVD risk, and to explore the factors associated with this early onset, thus helping to respond in the short term and to avoid the development of CVD in the long term. These equations take into account the main modifiable risk factors for CVD, such as high blood glucose levels, triglycerides, diastolic and systolic blood pressure (DBP/SBP), body mass index (BMI), waist and hip circumferences and low levels of HDL-c or high levels of LDL-c, to obtain a final score of cardiometabolic risk for each individual.

Diet is another modifiable risk factor of particular interest to public health in relation to cardiometabolic risk [3]. To date, a multitude of studies have focused on exploring the role of diet in CVD from a macronutrient-focused approach, such as low-fat or low-carb diets [4]. However, there is less evidence on the role of a food pattern as a whole, focusing on the consumption of foods and their interactions, and the relationship it could have with cardiovascular risk [5, 6]. The Mediterranean diet (MedDiet) pattern has been one of the most studied food patterns up to now. In a review of 27 studies published by Martínez-González et al., a higher adherence to the MedDiet pattern as measured by the Trichopoulou’s index showed an 11% reduction in the risk of cardiovascular events [7].

The vegetarian diet is another food pattern that has also been recognized for its beneficial effects on numerous health events, such as reducing morbidity including less risk of obesity, hypertension, or type 2 diabetes (T2D), among others, and mortality from chronic diseases [8–12]. This food pattern is characterized by the absence of some animal foods, such as red and processed meats, and a high consumption of plant-based foods, such as fruits, vegetables, legumes or nuts, which could explain its benefits. Thus, while the animal foods might play a harmful role because of their content in certain nutrients (e.g., saturated fat or heme iron), the plant-based foods may have a protective role through antioxidant nutrients (e.g., polyphenols) and fiber [13]. Hence the interest in knowing whether a pro-vegetarian (PVG) food pattern could act as an early marker of cardiometabolic risk may be well justified, especially in non-vegetarian populations. In a cross-sectional analysis of the PREDIMED study, a priori defined PVG index (gPVG) was developed based by positively scoring the consumption of plant-based foods and negatively the consumption of animal origin foods, in 7216 men and women aged 55–80 at high cardiovascular risk, showing a reduction in total mortality [14]. Since not all plant-based foods are equally healthy, Satija et al. subsequently proposed to differentiate between a healthful PVG food pattern (hPVG) which positively scores healthful plant-based foods (fruits, vegetables, legumes, whole grains, nuts, olive oil and coffee), and an unhealthful PVG food pattern (uPVG), which positively scores unhealthful plant-based foods, such as juices, chips, refined cereals, sugary drinks and pastries [15]. A more recently published study carried out with more than 70,000 U.S. women, found that those women with higher adherence to hPVG were less likely to develop coronary heart disease, while those with higher adherence to the uPVG showed a higher risk [16]. In the prospective follow-up study of the University of Navarra (SUN) with 11,554 participants an inverse association between adherence to a hPVG pattern and overweight and obesity was shown [17].

Therefore, it might be of interest to add evidence about the association of increased adherence to PVG patterns on early cardiovascular risk markers as measured by the standardized Metabolic Syndrome score (MetS z-score) and its components, in the context of the PREDIMED-Plus randomized intervention study, which includes participants at high cardiovascular risk and with a low prevalence of vegetarians. This would help to broaden our knowledge of the possible protective role of these food patterns and to propose more healthy dietary recommendations. Thus, the aim of this study was to explore the cross-sectional association between three plant-based diet patterns (gPVG, hPVG and uPVG) and MetS z-score, in the adult population of the PREDIMED-Plus study.

## Material and methods

### Study population

The present study is a cross-sectional assessment conducted within the PREDIMED-Plus project (Spain) (www.predimedplus.es). This intervention study aims to evaluate the effect of an intensive intervention with weight loss objectives based on the consumption of a low-calorie MedDiet, promotion of physical activity and behavioral therapy in the primary prevention of CVD and has been described in detail elsewhere [18]. Briefly, the participants included in this project were men (55–75 years) and women (60–75 years) with overweight or obesity (BMI 27–40 kg/m^2^) who meet at least three criteria of the Metabolic Syndrome (MetS) according to the updated criteria of the International Diabetes Federation and the American Heart Association and National Heart, Lung and Blood Institute [19] and without prior cardiovascular events.

**Fig. 1:**
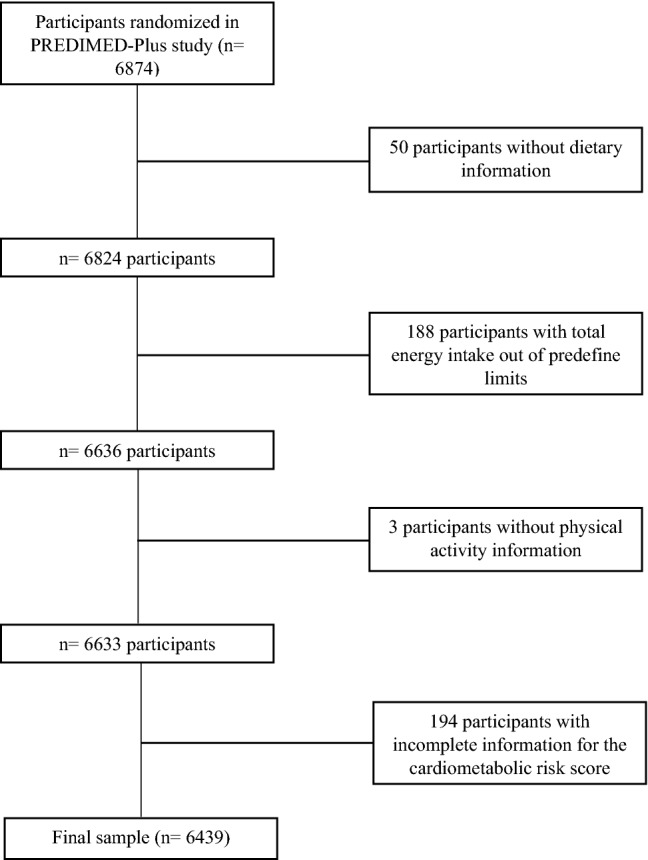
Flowchart of participants included in the present analysis from the PREDIMED-Plus Study

Recruitment of participants took place between September 2013 and December 2016 including 6874 participants who were randomized. After excluding participants with missing data for the dietary baseline information, for the parameters necessary to the calculation of MetS z-score and those with implausible values for the mean daily energy intake (< 500 and > 3500 kcal/day for women, < 800 and > 4000 kcal/day for men) [20], 6439 participants were included in the present study (Fig. 1). All participants signed the informed consent, and the project protocol was approved by the Research Ethics Committees from all recruiting centers according to the ethical standards of the Declaration of Helsinki. The trial was registered at the International Standard Randomized Controlled Trial (ISRCTN:http://www.isrctn.com/ISRCTN89898870).

### Dietary assessment and pro-vegetarian food patterns

To obtain the final score of the different PVG patterns, the dietary information was evaluated using a semi-quantitative food frequency questionnaire (FFQ) previously validated in Spain [21, 22]. The FFQ was completed at a baseline visit with the help of a trained interviewer. The FFQ includes a list of 143 foods specifying the standard size or ration of consumption over a period of the previous year including 9 possible responses to determine the frequency of consumption ranging from “never or < 1 month” to “ ≥ 6 times a day”.

For the creation of the gPVG pattern, the methodology proposed by Martínez-González [14] was followed. For healthful and unhealthful PVG versions, the method proposed by Satija et al. [15] was the reference. Dietary information from 18 food groups (Vegetables, Fruits, Legumes, Whole Grains, Refined Grains, Cooked or Roasted Potatoes, Chips, Nuts, Olive Oil, Tea and Coffee, Fruit Juices, Sugary Drinks, Sweets and Desserts, Meat and Meat Products, Animal Fats, Eggs, Fish and Seafood and Dairy) was used. Table 1 specifies the items included in the 18 food groups and the scoring criteria for each pattern.

**Table 1:** Scoring criteria for the PVG food patterns^a^

Component	Included foods	gPVG^c^	hPVG	uPVG
Plant food groups^b^				
1. Vegetables	Swiss chard, spinach, cauliflower, broccoli, lettuce, tomatoes, carrot, green beans, zucchini, eggplant, cucumber, peppers, asparagus, onion, other fresh vegetables	Positive	Positive	Reverse
2. Fruits	Citrus, banana, apple, pear, strawberry, cherry, peach, fig, melon, watermelon, grapes, kiwi, canned fruit	Positive	Positive	Reverse
3. Legumes	Lentils, beans, chickpeas, peas	Positive	Positive	Reverse
4. Whole grains	Whole-grain bread, muesli, brown rice, whole-grain pasta	Positive	Positive	Reverse
5. Refined grains	White bread, breakfast cereals, white rice, white pasta	Positive	Reverse	Positive
6. Potatoes*	Potato chips, French fries, boiled potatoes	Positive	Reverse	Positive
7. Nuts	Almonds, pistachios, walnuts, other nuts	Positive	Positive	Reverse
8. Olive oil	Refined olive oil, extra-virgin olive oil, olive pomace oil	Positive	Positive	Reverse
9. Tea and coffee	Caffeinated coffee, decaffeinated coffee, tea	Not scored	Positive	Reverse
10. Fruit juices	Orange juice, other natural fruits juice	Not scored	Reverse	Positive
11. Sugar-sweetened beverages	Regular soft drinks, low calorie soft drinks, fruit flavored punch or noncarbonated beverages	Not scored	Reverse	Positive
12. Sweets and desserts	Cookies, chocolate cookies, whole-grain cookies, home-made cakes and biscuits, croissant, tea pastries, industrial cakes, donuts, cupcake, muffin, chocolate, cocoa powder, nougat, marzipan, sugar	Not scored	Reverse	Positive
Animal food groups				
13. Meat/meat products	Chicken or turkey with or without skin, beef, pork, lamb, rabbit, liver, viscera, Parma ham, cooked ham, cured meats, salami, mortadella, spicy pork sausage, hot dogs, foie gras, hamburger, bacon	Reverse	Reverse	Reverse
14. Animal fats for cooking or as a spread	Butter, lard	Reverse	Reverse	Reverse
15. Eggs	Eggs	Reverse	Reverse	Reverse
16. Fish and other seafood	White fish, blue fish, salad or smoked fish, clams, squid, shrimp, oil canned fish, natural canned fish	Reverse	Reverse	Reverse
17. Dairy products	Whole milk, skim or low-fat milk, condensed milk, cream, milk shake, full fat yogurt, low-fat yogurt, cheese, custard, ice cream	Reverse	Reverse	Reverse

^a^Positive indicates that higher consumption of this food group received higher scores. Reverse indicates that higher consumption of this food group received lower scores

^b^In the hPVG food pattern, whole grains, fruits, vegetables, nuts, legumes, potatoes* (boiled), tea, and coffee were considered “healthy plant foods.” Refined grains, French fries and chips*, fruit juices, sugar-sweetened and artificially sweetened beverages, and sweets and desserts were considered “unhealthy plant foods.” The gPVG food pattern did not differentiate plant foods as healthy or unhealthy

^c^In the gPVG food pattern, consumption of whole grains and refined grains was aggregated as the “grains” food group

In short, to create the different PVG food patterns, consumption in grams of the 18 food groups was adjusted for total energy intake following the residual method [23]. After that, calorie-adjusted consumption in grams was categorized into quintiles giving values of 1–5 according to the consumption quintile of each food group. In the case of the gPVG food pattern seven components, belonging to the plant food groups, scored positively: vegetables, fruits, legumes, grains (whole and refined), potatoes (cooked, roasted and/or fried), nuts and olive oil, and five components (meat and other products, animal fats, eggs, seafood, and dairy), belonging to animal food groups were scored reversely (a value of 5 for lowest consumption). For the hPVG and uPVG, the grain group was separated into whole and refined grains and the potatoes group in fried or chips and cooked or roasted. Four new groups (tea and coffee, natural fruit juices, sweetened drinks and desserts or sweets) were also introduced in both, hPVG and uPVG. To obtain the score of each participant, the points for the 12 components, in the case of the gPVG pattern, and for the 18 groups, in the case of the hPVG and uPVG patterns, were be sum. So, the possible results ranged from 12 points (minimum adherence) to 60 points (maximum adherence) for the gPVG pattern, and from 18 points (minimum adherence) to 90 points (maximum adherence) for the hPVG and uPVG patterns.

### MetS z-score and its components

The continuous cardiometabolic risk scale that we used was the MetS z-score proposed by Franks [24]. Prior to the calculation of this scale, all variables were standardized for the total number of participants, except for HDL and waist to hip ratio (WHR) which were standardized by sex using sex-specific cut-off points. The original version of MetS z-score includes fasting insulin in the formula, but we exclude that parameter from the calculation since it was not measured and determined. We also calculated standardized components of MetS z-score (BMI, WHR, SBP/DBP, HDL-c, plasma triglycerides and plasma glucose).

Weight, height, waist and hip circumference were measured by duplicated with light clothing and no shoes using a calibrate scale, a wall-mounted stadiometer, and a non-elastic tape, respectively. Waist circumference was measured midway between the lowest rib and the iliac crest. Hip circumference was measured at the widest part. BMI was calculated as weight (kg) divided by height (meters) squared, and WHR as waist circumference (in cm) divided by hip circumference (in cm). Blood pressure was measured three times with a validated semiautomatic oscillometer after 5 min of rest in-between measurement (Omron HEM-705CP, Hoofddorp, The Netherlands), and the mean of the three measurements was used. After an overnight fast, blood samples were collected at baseline and aliquots of serum and ethylene diamine tetraacetic acid (EDTA) plasma were immediately processed, coded and stored at − 80 °C in a central laboratory until analysis. High Density Lipoprotein (HDL), serum glucose and triglyceride levels were determined by standard enzymatic methods in automatic analyzers in local laboratories.

The MetS z-score for each participant was obtained using the following formula:

(BMI + WHR)/2 + (SBP + DBP)/2 + hyperglycemia (plasma fasting glucose)—HDLc + triglycerides

### Covariates

Other sociodemographic variables, lifestyles and previous history of various diseases, as well as assigned intervention, was also collected at baseline. Information about total physical activity in Metabolic Equivalents (METS) min/day was measured using the validated Regicor Short Physical Activity Questionnaire [25]. Adherence to MedDiet was valued with a 17-item questionnaire, a modified version of a previously validated 14-item questionnaire [26], for an energy-restricted version.

### Statistical analysis

Descriptive analysis of participants’ characteristics according to quintiles of each PVG food pattern adherence was shown as mean and standard deviation (SD) for quantitative traits, and percentage for categorical variables. We performed the ANOVA test for quantitative variables and the Chi-square test for qualitative variables to compare the characteristics of the sample between adherence quintiles.

Multiple robust linear regression models were performed using an MM-type estimator by adjusting for possible confounders to explore the association between adherence to each PVG food pattern (in quintiles and per 5 points increment in adherence) and MetS z-score, along and with its components separately [27]. Regression coefficients represent the change in each outcome, where 1 unit is equivalent to a 1-SD difference in z scores, or a 1-unit difference in the MetS z-score or its components, per one point of dietary adherence to PVG food patterns, either in the continuous (per each 5 points of adherence) or quintiles form of the different PVG food patterns.

Possible confounder selection was based on a previous review of the literature. It was also adjusted by those variables that when estimating the effect of exposure, the effect changed by ≥ 10% when excluding the variable from the model. Crude model was minimally adjusted for energy intake. Model 1 was additionally adjusted for age (continuous) and sex. Model 2 was additionally adjusted for educational level (illiterate or primary education, secondary education, academic or graduate, and missing information), smoking status (current smoker, former smoker, and never smoker), alcohol intake (grams/day) and total physical activity per day (METS-min/day).

Statistical analyses were carried out with R 3.5.1 (R Foundation for Statistical Computing, Vienna, Austria; http://www.R-project.org). For robust linear regression analyses, we also used a robust base package of statistical software R. We used the database version of the PREDIMED-Plus dated March 2019.

## Results

Baseline characteristics of participants according to quintiles of the three PVG food patterns are presented in Table 2. Participants with a higher adherence to gPVG and hPVG patterns were more likely to be older, more physically active, have a lower BMI and better adhere to the MedDiet pattern. Inversely, those participants with a higher adherence to the uPVG pattern were more likely to be younger, smoker, less physically active and less adherent to the MedDiet. Lower education and lower alcohol consumption were observed in those participants with higher adherence to the gPVG pattern, and a higher alcohol consumption in more adhered participants to hPVG and uPVG patterns. Diabetes prevalence was lower in participants with a higher adherence to uPVG pattern.

**Table 2:** Baseline characteristics of participants according to quintiles of the three PVG food patterns: the PREDIMED-Plus Study (*n* = 6439)

	gPVG food pattern^a^				
	Very low: < 33 (*n* = 1589)	Low: 33–35 (*n* = 1345)	Moderate: 36–37 (*n* = 980)	High: 38–40 (*n* = 1297)	Very High: > 40 (*n* = 1228)
Sex, male (%)	52.1	52.2	51.0	50.9	52.1
Age (y)	64.4 (5.0)^b^	64.7 (4.8)	65.2 (4.9)	65.4 (4.9)	65.5 (4.9)
Illiterate or primary education (%)	44.6	48.0	50.3	52.4	52.6
Hypertension (%)	83.8	82.8	84.4	82.7	84.0
High blood cholesterol (%)	69.4	70.3	67.6	70.0	69.3
Diabetes (%)	31.4	32.6	32.4	28.0	29.7
BMI (Kg/m^2^)	32.8 (3.5)	32.8 (3.5)	32.4 (3.4)	32.3 (3.5)	32.3 (3.3)
Smoking (% current smokers)	14.7	11.6	11.6	12.6	12.7
Alcohol intake (g/d)	11.8 (15.6)	11.5 (15.1)	10.6 (15.0)	11.0 (15.1)	10.3 (14.4)
Physical activity (MET-min/d)^c^	326.3 (311.3)	332.3 (330.4)	357.6 (314.8)	360.6 (335.5)	394.0 (353.4)
Adherence to Mediterranean diet (0–17 points)^d^	7.8 (2.6)	8.3 (2.6)	8.6 (2.5)	8.8 (2.6)	9.3 (2.7)

	hPVG food pattern				
	Very low: < 49 (*n* = 1454)	Low: 49–52 (*n* = 1231)	Moderate: 53–56 (*n* = 1391)	High: 57–60 (*n* = 1241)	Very high: > 60 (*n* = 1122)
Sex, male (%)	52.3	49.3	51.8	54.4	50.6
Age (y)	64.5 (5.0)	64.7 (4.9)	65.1 (4.8)	65.4 (4.9)	65.5 (4.8)
Illiterate or primary education (%)	49.1	50.0	50.1	48.5	48.7
Hypertension (%)	84.4	84.0	83.9	83.5	81.2
High blood cholesterol (%)	70.2	69.5	69.7	67.8	69.9
Diabetes (%)	32.1	29.7	29.2	32.2	30.8
BMI (Kg/m^2^)	32.8 (3.5)	32.6 (3.4)	32.5 (3.4)	32.3 (3.4)	32.4 (3.4)
Smoking (% current smokers)	14.1	13.7	11.4	12.3	12.2
Alcohol intake (g/d)	9.9 (13.3)	10.4 (14.6)	11.6 (15.8)	11.6 (15.2)	12.3 (16.6)
Physical activity (MET-min/d)	303.9 (307.1)	332.5 (315.0)	348.1 (319.6)	379.1 (335.0)	411.4 (367.8)
Adherence to Mediterranean diet (0–17 points)^d^	7.2 (2.4)	8.2 (2.5)	8.5 (2.4)	9.1 (2.5)	10.0 (2.6)

	uPVG food pattern				
	Very low: < 49 (*n* = 1504)	Low: 49–52 (*n* = 1197)	Moderate: 53–56 (*n* = 1288)	High: 57–60 (*n* = 1192)	Very high: > 60 (*n* = 1258)
Sex, male (%)	51.1	51.5	51.6	53.9	50.7
Age (y)	65.7 (4.9)	64.7 (4.9)	65.1 (4.9)	64.7 (4.8)	64.6 (5.0)
Illiterate or primary education (%)	48.1	48.0	49.5	49.8	51.2
Hypertension (%)	82.2	84.0	84.6	83.7	83.0
High blood cholesterol (%)	69.9	67.8	69.8	70.1	69.2
Diabetes (%)	37.3	30.2	31.1	26.8	27.0
BMI (Kg/m^2^)	32.4 (3.4)	32.5 (3.5)	32.6 (3.4)	32.6 (3.4)	32.6 (3.5)
Smoking (% current smoker)	10.4	11.7	13.5	13.2	15.6
Alcohol intake (g/d)	8.6 (11.8)	10.8 (14.0)	11.6 (15.8)	12.3 (16.4)	12.7 (17.0)
Physical activity (MET-min/d)	396.7 (344.6)	374.6 (356.3)	352.5 (320.1)	330.5 (306.6)	297.7 (307.1)
Adherence to Mediterranean diet (0–17 points)^d^	10.0 (2.5)	9.1 (2.5)	8.5 (2.5)	7.7 (2.3)	7.0 (2.4)

^a^Comparisons of characteristics across quintiles of the PVG food patterns were performed using 1-factor ANOVA for quantitative variables or chi-square tests for categorical variables

^b^Mean (SD) (all such values)

^c^*MET-min* metabolic equivalent task minutes

^d^Adherence to an energy-restricted MedDiet was assessed using a 17-item questionnaire, a modified version of a validated 14-item questionnaire [26]

The results of the multiple robust linear regression analysis for the association between the different PVG patterns (in quintiles of adherence and in continuous for every 5 points) and the score of MetS with its components separately are presented in Tables 3, 4 and 5. Reduction in MetS score and its components separately is shown in units of SD according to quintiles of adherence for the PVG food patterns (*p* trend < 0.001). After adjusting for energy intake, sex, age, educational level, smoking status, alcohol intake and total physical activity per day, we observed a reduction in the global MetS z score (regression coefficient, ‘β’ for fifth quintile (Q5) vs. first quintile (Q1) =− 0.16; 95% CI: − 0.33 to 0.01; *p*-trend: 0.015), the BMI (β for Q5 vs Q1 =− 0.14; 95% CI: − 0.22 to − 0.06; *p* trend: < 0.001) and the WHR (β for Q5 vs Q1 =− 0.16; 95% CI: − 0.23 to − 0.09; *p* trend: < 0.001) in those participants with “very high” adherence (> 40 points) to the gPVG pattern (Table 3). Also, we observed direct associations with DBP (β for Q5 vs Q1 = 0.11; 95% CI: 0.03 to 0.18; *p* trend: 0.009) and HDL-cholesterol (β for Q5 vs Q1 = 0.07; 95% CI: 0.00 to 0.14; *p* trend: 0.046) in the fully adjusted gPVG food pattern model. When the gPVG pattern was considered as continuous variable (every 5 points of increment) we observed inverse associations with BMI *β* = − 0.06 (95% CI: − 0.09; − 0.04) and WHR *β* = − 0.06 (95% CI: − 0.08; − 0.03). On the other hand, we observed a direct association with DBP *β* = 0.03 (95% CI: 0.01; 0.06).

**Table 3:** Association between adherence to general PVG food pattern (β^a^ and 95% confidence intervals for pattern in quintiles and continuous per 5-units) and metabolic syndrome z-score and its components at baseline in participants PREDIMED-Plus Study (*n* = 6439)

	gPVG food pattern quintile						
	Very low: < 33 (*n* = 1589)	Low: 33–35 (*n* = 1345)	Moderate: 36–37 (*n* = 980)	High: 38–40 (*n* = 1297)	Very high: > 40 (*n* = 1228)	*p* trend	Per 5 points increment in adherence
Metabolic syndrome z-score^b^							
Crude	Ref	− 0.02 (− 0.19; 0.14)	− 0.06 (− 0.24; 0.13)	− 0.23 (− 0.40; − 0.06)	− 0.23 (− 0.40; − 0.06)	0.001	− 0.08 (− 0.14; − 0.03)
Multiple adjusted 1	Ref	0.00 (− 0.17; 0.17)	− 0.01 (− 0.20; 0.17)	− 0.18 (− 0.35; − 0.01)	− 0.16 (− 0.33; 0.01)	0.016	− 0.06 (− 0.11; 0.00)
Multiple adjusted 2	Ref	− 0.02 (− 0.19; 0.15)	− 0.02 (− 0.20; 0.17)	− 0.20 (− 0.37; − 0.03)	− 0.16 (− 0.33; 0.01)	0.015	− 0.06 (− 0.12; 0.00)
Body mass index^b^							
Crude	Ref	0.01 (− 0.07; 0.09)	− 0.10 (− 0.19; − 0.02)	− 0.14 (− 0.22; − 0.06)	− 0.15 (− 0.23; -0.07)	< 0.001	− 0.07 (− 0.09; − 0.04)
Multiple adjusted 1	Ref	0.01 (− 0.07; 0.09)	− 0.10 (− 0.19; − 0.02)	− 0.14 (− 0.22; − 0.06)	− 0.15 (− 0.23; − 0.07)	< 0.001	− 0.07 (− 0.09; − 0.04)
Multiple adjusted 2	Ref	0.00 (− 0.08; 0.08)	− 0.11 (− 0.19; − 0.02)	− 0.14 (− 0.22; − 0.06)	− 0.14 (− 0.22; − 0.06)	< 0.001	− 0.06 (− 0.09; − 0.04)
Waist-to-hip ratio^c^							
Crude	Ref	− 0.04 (− 0.12; 0.03)	− 0.07 (− 0.14; 0.01)	− 0.10 (− 0.18; − 0.03)	− 0.15 (− 0.22; − 0.07)	< 0.001	− 0.05 (− 0.07; − 0.03)
Multiple adjusted 1	Ref	− 0.05 (− 0.12; 0.03)	− 0.08 (− 0.15; 0.00)	− 0.11 (− 0.19; − 0.04)	− 0.16 (− 0.23; − 0.09)	< 0.001	− 0.06 (− 0.08; − 0.03)
Multiple adjusted 2	Ref	− 0.05 (− 0.12; 0.02)	− 0.08 (− 0.15; 0.00)	− 0.12 (− 0.19; − 0.04)	− 0.16 (− 0.23; − 0.09)	< 0.001	− 0.06 (− 0.08; − 0.03)
Systolic blood pressure							
Crude	Ref	0.03 (− 0.05; 0.10)	0.05 (− 0.03; 0.13)	0.01 (− 0.06; 0.08)	0.03 (− 0.05; 0.10)	0.588	0.01 (− 0.01; 0.03)
Multiple adjusted 1	Ref	0.02 (− 0.05; 0.09)	0.04 (− 0.04; 0.11)	0.00 (− 0.08; 0.07)	0.01 (− 0.06; 0.09)	0.910	0.00 (− 0.02; 0.03)
Multiple adjusted 2	Ref	0.02 (− 0.05; 0.09)	0.05 (− 0.03; 0.13)	0.00 (− 0.07; 0.08)	0.03 (− 0.05; 0.10)	0.606	0.01 (− 0.02; 0.03)
Diastolic blood pressure^b^							
Crude	Ref	0.05 (− 0.02; 0.13)	0.02 (− 0.06; 0.10)	0.02 (− 0.06; 0.09)	0.01 (− 0.06; 0.09)	0.935	0.00 (− 0.02; 0.03)
Multiple adjusted 1	Ref	0.08 (0.00; 0.15)	0.07 (− 0.01; 0.15)	0.08 (0.00; 0.15)	0.09 (0.02; 0.17)	0.023	0.03 (0.00; 0.05)
Multiple adjusted 2	Ref	0.08 (0.01; 0.15)	0.07 (− 0.01; 0.15)	0.08 (0.01; 0.16)	0.11 (0.03; 0.18)	0.009	0.03 (0.01; 0.06)
HDL-cholesterol^c^							
Crude	Ref	0.03 (− 0.04; 0.10)	0.01 (− 0.07; 0.08)	0.04 (− 0.03; 0.12)	0.05 (− 0.02; 0.12)	0.137	0.01 (− 0.02; 0.03)
Multiple adjusted 1	Ref	0.03 (− 0.04; 0.09)	0.00 (− 0.07; 0.08)	0.03 (− 0.04; 0.11)	0.04 (− 0.03; 0.11)	0.277	0.00 (− 0.02; 0.02)
Multiple adjusted 2	Ref	0.03 (− 0.03; 0.10)	0.01 (− 0.06; 0.09)	0.06 (− 0.02; 0.13)	0.07 (0.00; 0.14)	0.046	0.01 (− 0.01; 0.04)
Plasma triglycerides^b^							
Crude	Ref	0.01 (− 0.05; 0.06)	0.00 (− 0.06; 0.06)	0.01 (− 0.04; 0.06)	− 0.01 (− 0.06; 0.04)	0.805	0.00 (− 0.01; 0.02)
Multiple adjusted 1	Ref	0.01 (− 0.04; 0.06)	0.01 (− 0.05; 0.07)	0.02 (− 0.03; 0.08)	0.01 (− 0.05; 0.06)	0.688	0.01 (− 0.01; 0.03)
Multiple adjusted 2	Ref	0.01 (− 0.04; 0.07)	0.02 (− 0.04; 0.08)	0.03 (− 0.02; 0.09)	0.02 (− 0.04; 0.07)	0.379	0.01 (0.00; 0.03)
Plasma glucose^b^							
Crude	Ref	− 0.01 (− 0.06; 0.04)	− 0.01 (− 0.06; 0.04)	− 0.06 (− 0.10; − 0.01)	− 0.02 (− 0.07; 0.03)	0.150	− 0.01 (-0.03; 0.01)
Multiple adjusted 1	Ref	− 0.01 (− 0.06; 0.04)	− 0.01 (− 0.06; 0.04)	− 0.05 (− 0.10; − 0.01)	− 0.02 (− 0.07; 0.03)	0.174	− 0.01 (− 0.03; 0.01)
Multiple adjusted 2	Ref	− 0.01 (− 0.06; 0.04)	− 0.01 (− 0.06; 0.04)	− 0.06 (− 0.10; − 0.01)	− 0.01 (− 0.07; 0.04)	0.228	− 0.01 (− 0.02; 0.01)

*HDL-c* high-density lipoprotein-cholesterol

^a^MM-type estimators for linear robust regression models, the betas represent the change in each outcome, where 1 unit is equivalent to a 1-SD difference in z scores, or a 1-unit difference in the MetS z-score or its components, per one point of dietary adherence to PVG food patterns, either in the continuous (per each 5 points of adherence) or quintiles form of the different PVG food patterns

^b^Data were standardized

^c^*p trend* test for linear trend were conducted using the adherence to a gPVG food pattern quintile, *Crude* adjusted for energy intake, *multiple adjusted 1* additionally adjusted for sex and age, *multiple adjusted 2* additionally adjusted for educational level, *smoking status* alcohol intake, and total physical activity per day

**Table 4:** Association between adherence for healthful PVG food pattern (β^a^ and 95% confidence intervals for pattern in quintiles and continuous, per 5-units) and metabolic syndrome z-score and its components at baseline in participants PREDIMED-Plus Study (*n* = 6439)

	hPVG food pattern quintile						
	Very low: < 49 (*n* = 1454)	Low: 49–52 (*n* = 1231)	Moderate: 53–56 (*n* = 1391)	High: 57–60 (*n* = 1241)	Very high: > 60 (*n* = 1122)	*p* trend	Per 5 points increment in adherence
Metabolic syndrome z-score^b^							
Crude	Ref	− 0.25 (− 0.43; − 0.08)	− 0.32 (− 0.49; − 0.15)	− 0.29 (− 0.47; − 0.12)	− 0.40 (− 0.58; − 0.22)	< 0.001	− 0.10 (− 0.14; − 0.06)
Multiple adjusted 1	Ref	− 0.23 (− 0.41; − 0.06)	− 0.28 (− 0.45; − 0.11)	− 0.24 (− 0.42; − 0.07)	− 0.32 (− 0.50; − 0.14)	< 0.001	− 0.08 (− 0.12; − 0.04)
Multiple adjusted 2	Ref	− 0.22 (− 0.39; − 0.04)	− 0.24 (− 0.41; − 0.07)	− 0.21 (− 0.39; − 0.03)	− 0.23 (− 0.41; − 0.05)	0.016	− 0.06 (− 0.10; − 0.02)
Body mass index^b^							
Crude	Ref	− 0.07 (− 0.15; 0.01)	− 0.10 (− 0.18; − 0.02)	− 0.15 (− 0.23; − 0.07)	− 0.11 (− 0.20; − 0.02)	0.002	− 0.03 (− 0.05; − 0.01)
Multiple adjusted 1	Ref	− 0.08 (− 0.16; 0.00)	− 0.11 (− 0.18; − 0.03)	− 0.14 (− 0.23; − 0.06)	− 0.12 (− 0.20; − 0.03)	0.002	− 0.03 (− 0.05; − 0.01)
Multiple adjusted 2	Ref	− 0.07 (− 0.15; 0.01)	− 0.10 (− 0.17; − 0.02)	− 0.12 (− 0.20; − 0.04)	− 0.07 (− 0.15; 0.02)	0.043	− 0.02 (− 0.04; 0.00)
Waist-to-hip ratio^c^							
Crude	Ref	− 0.06 (− 0.14; 0.01)	− 0.07 (− 0.15; 0.00)	− 0.10 (− 0.17; − 0.02)	− 0.15 (− 0.22; − 0.07)	< 0.001	− 0.04 (− 0.05; − 0.02)
Multiple adjusted 1	Ref	− 0.06 (− 0.14; 0.01)	− 0.08 (− 0.16; − 0.01)	− 0.11 (− 0.18; − 0.03)	− 0.16 (− 0.24; − 0.09)	< 0.001	− 0.04 (− 0.06; − 0.03)
Multiple adjusted 2	Ref	− 0.06 (− 0.14; 0.01)	− 0.08 (− 0.15; 0.00)	− 0.10 (− 0.18; − 0.03)	− 0.14 (− 0.22; − 0.07)	< 0.001	− 0.04 (− 0.05; − 0.02)
Systolic blood pressure^b^							
Crude	Ref	− 0.02 (− 0.10; 0.05)	0.03 (− 0.05; 0.11)	0.01 (− 0.06; 0.09)	0.01 (− 0.07; 0.09)	0.564	0.00 (− 0.02; 0.02)
Multiple adjusted 1	Ref	− 0.02 (− 0.09; 0.06)	0.02 (− 0.05; 0.10)	0.00 (− 0.08; 0.07)	0.01 (− 0.07; 0.08)	0.818	0.00 (− 0.02; 0.01)
Multiple adjusted 2	Ref	− 0.01 (− 0.09; 0.06)	0.02 (− 0.05; 0.10)	0.00 (− 0.07; 0.08)	0.01 (− 0.07; 0.09)	0.721	0.00 (− 0.02; 0.02)
Diastolic blood pressure^b^							
Crude	Ref	− 0.08 (− 0.16; 0.00)	− 0.06 (− 0.14; 0.02)	− 0.07 (− 0.15; 0.00)	− 0.10 (− 0.18; − 0.02)	0.020	− 0.03 (− 0.05; − 0.01)
Multiple adjusted 1	Ref	− 0.05 (− 0.13; 0.03)	− 0.01 (− 0.09; 0.06)	− 0.01 (− 0.09; 0.06)	− 0.01 (− 0.09; 0.07)	0.966	0.00 (− 0.02; 0.01)
Multiple adjusted 2	Ref	− 0.05 (− 0.12; 0.03)	− 0.01 (− 0.08; 0.07)	− 0.01 (− 0.09; 0.07)	0.00 (− 0.08; 0.08)	0.785	0.00 (− 0.02; 0.02)
HDL− cholesterol^c^							
Crude	Ref	− 0.02 (− 0.09; 0.05)	0.06 (− 0.01; 0.12)	0.07 (− 0.01; 0.14)	0.08 (0.01; 0.16)	0.006	0.02 (0.01; 0.04)
Multiple adjusted 1	Ref	− 0.02 (− 0.10; 0.05)	0.05 (− 0.02; 0.12)	0.06 (− 0.02; 0.13)	0.07 (− 0.01; 0.15)	0.021	0.02 (0.00; 0.04)
Multiple adjusted 2	Ref	− 0.03 (− 0.10; 0.04)	0.03 (− 0.03; 0.10)	0.04 (− 0.03; 0.12)	0.05 (− 0.02; 0.13)	0.068	0.01 (0.00; 0.03)
Plasma triglycerides^b^							
Crude	Ref	− 0.06 (− 0.11; 0.00)	− 0.07 (− 0.13; − 0.02)	− 0.04 (− 0.10; 0.02)	− 0.09 (− 0.15; − 0.04)	0.004	− 0.02 (− 0.03; − 0.01)
Multiple adjusted 1	Ref	− 0.05 (− 0.11; 0.01)	− 0.06 (− 0.12; − 0.01)	− 0.03 (− 0.08; 0.03)	− 0.08 (− 0.14; − 0.02)	0.029	− 0.01 (− 0.03; 0.00)
Multiple adjusted 2	Ref	− 0.05 (− 0.11; 0.01)	− 0.05 (− 0.11; 0.00)	− 0.02 (− 0.07; 0.04)	− 0.06 (− 0.11; 0.00)	0.165	− 0.01 (− 0.02; 0.00)
Plasma glucose^b^							
Crude	Ref	− 0.02 (− 0.07; 0.03)	0.00 (− 0.05; 0.05)	0.03 (− 0.02; 0.08)	0.01 (− 0.04; 0.07)	0.250	0.01 (0.00; 0.02)
Multiple adjusted 1	Ref	− 0.02 (− 0.07; 0.03)	0.00 (− 0.04; 0.05)	0.03 (− 0.02; 0.09)	0.02 (− 0.03; 0.07)	0.189	0.01 (0.00; 0.02)
Multiple adjusted 2	Ref	− 0.02 (− 0.07; 0.03)	0.01 (− 0.04; 0.05)	0.04 (− 0.01; 0.09)	0.03 (− 0.02; 0.08)	0.110	0.01 (0.00; 0.02)

*HDL-c* high-density lipoprotein-cholesterol

^a^MM-type estimators for linear robust regression models, the betas represent the change in each outcome, where 1 unit is equivalent to a 1-SD difference in z scores, or a 1-unit difference in the MetS z-score or its components, per one point of dietary adherence to PVG food patterns, either in the continuous (per each 5 points of adherence) or quintiles form of the different PVG food patterns

^b^Data were standardized

^c^*p* trend test for linear trend were conducted using the adherence to a hPVG food pattern quintile, *Crude* adjusted for energy intake, *multiple adjusted 1* additionally adjusted for sex and age, *multiple adjusted 2* additionally adjusted for educational level, smoking status, alcohol intake, and total physical activity per day

**Table 5:** Association between adherence for unhealthful PVG food pattern (β^a^ and 95% confidence intervals for pattern in quintiles and continuous, per 5-units) and metabolic syndrome z-score and its components at baseline in participants PREDIMED-Plus Study (*n* = 6439)

	uPVG food pattern quintile						
	Very low: < 49 (*n* = 1504)	Low: 49–52 (*n* = 1197)	Moderate: 53–56 (*n* = 1288)	High: 57–60 (*n* = 1192)	Very high: > 60 (*n* = 1258)	*p* trend	Per 5 points increment in adherence
Metabolic syndrome z-score^b^							
Crude	Ref	− 0.03 (− 0.20; 0.14)	0.21 (0.04; 0.38)	0.07 (− 0.10; 0.24)	0.27 (0.10; 0.44)	0.001	0.06 (0.02; 0.09)
Multiple adjusted 1	Ref	− 0.05 (− 0.22; 0.12)	0.20 (0.03; 0.37)	0.05 (− 0.13; 0.22)	0.26 (0.09; 0.43)	0.002	0.05 (0.02; 0.09)
Multiple adjusted 2	Ref	− 0.04 (− 0.21; 0.13)	0.19 (0.02; 0.36)	0.01 (− 0.16; 0.19)	0.21 (0.04; 0.38)	0.019	0.04 (0.00; 0.07)
Body mass index^b^							
Crude	Ref	0.03 (− 0.05; 0.11)	0.06 (− 0.02; 0.14)	0.05 (− 0.03; 0.13)	0.04 (− 0.04; 0.12)	0.299	0.01 (− 0.01; 0.03)
Multiple adjusted 1	Ref	0.02 (− 0.06; 0.10)	0.04 (− 0.03; 0.12)	0.04 (− 0.04; 0.12)	0.02 (− 0.07; 0.10)	0.586	0.01 (− 0.01; 0.02)
Multiple adjusted 2	Ref	0.01 (− 0.07; 0.09)	0.02 (− 0.06; 0.10)	0.00 (− 0.08; 0.08)	− 0.02 (− 0.11; 0.06)	0.558	− 0.01 (− 0.02; 0.01)
Waist-to-hip ratio^c^							
Crude	Ref	− 0.04 (− 0.12; 0.03)	0.06 (− 0.01; 0.14)	0.04 (− 0.03; 0.12)	0.06 (− 0.01; 0.14)	0.028	0.02 (0.00; 0.03)
Multiple adjusted 1	Ref	− 0.03 (− 0.11; 0.04)	0.07 (0.00; 0.14)	0.05 (− 0.03; 0.12)	0.07 (0.00; 0.15)	0.017	0.02 (0.00; 0.04)
Multiple adjusted 2	Ref	− 0.04 (− 0.11; 0.04)	0.05 (− 0.02; 0.12)	0.03 (− 0.05; 0.10)	0.05 (− 0.03; 0.12)	0.104	0.01 (0.00; 0.03)
Systolic blood pressure^b^							
Crude	Ref	0.03 (− 0.04; 0.11)	0.04 (− 0.03; 0.12)	0.02 (− 0.06; 0.10)	− 0.02 (− 0.09; 0.06)	0.649	− 0.01 (− 0.02; 0.01)
Multiple adjusted 1	Ref	0.06 (− 0.02; 0.13)	0.06 (− 0.02; 0.13)	0.04 (− 0.03; 0.12)	0.02 (− 0.06; 0.09)	0.713	0.00 (− 0.01; 0.02)
Multiple adjusted 2	Ref	0.06 (− 0.02; 0.13)	0.06 (− 0.02; 0.13)	0.03 (− 0.05; 0.10)	0.01 (− 0.07; 0.08)	0.957	0.00 (− 0.02; 0.02)
Diastolic blood pressure^b^							
Crude	Ref	0.10 (0.03; 0.17)	0.07 (0.00; 0.14)	0.12 (0.04; 0.19)	0.12 (0.04; 0.20)	0.002	0.03 (0.01; 0.04)
Multiple adjusted 1	Ref	0.07 (0.00; 0.15)	0.06 (− 0.01; 0.13)	0.09 (0.01; 0.16)	0.09 (0.01; 0.16)	0.020	0.02 (0.00; 0.04)
Multiple adjusted 2	Ref	0.07 (− 0.01; 0.14)	0.06 (− 0.01; 0.13)	0.08 (0.00; 0.15)	0.08 (0.00; 0.15)	0.042	0.02 (0.00; 0.03)
HDL-cholesterol^c^							
Crude	Ref	− 0.02 (− 0.09; 0.05)	− 0.06 (− 0.12; 0.01)	− 0.06 (− 0.13; 0.01)	− 0.10 (− 0.17; − 0.03)	0.002	− 0.02 (− 0.04; 0.00)
Multiple adjusted 1	Ref	− 0.02 (− 0.09; 0.05)	− 0.05 (− 0.12; 0.02)	− 0.05 (− 0.12; 0.02)	− 0.10 (− 0.17; − 0.03)	0.005	− 0.02 (− 0.03; 0.00)
Multiple adjusted 2	Ref	− 0.03 (− 0.10; 0.04)	− 0.07 (− 0.14; 0.00)	− 0.07 (− 0.14; 0.00)	− 0.11 (− 0.18; − 0.04)	0.001	− 0.02 (− 0.04; − 0.01)
Plasma triglycerides^b^							
Crude	Ref	0.00 (− 0.06; 0.05)	0.06 (0.01; 0.11)	0.06 (0.00; 0.11)	0.10 (0.05; 0.15)	< 0.001	0.03 (0.01; 0.04)
Multiple adjusted 1	Ref	− 0.01 (− 0.07; 0.04)	0.05 (0.00; 0.11)	0.05 (0.00; 0.11)	0.09 (0.04; 0.15)	< 0.001	0.02 (0.01; 0.04)
Multiple adjusted 2	Ref	− 0.01 (− 0.07; 0.04)	0.05 (0.00; 0.10)	0.04 (− 0.02; 0.09)	0.08 (0.02; 0.13)	0.003	0.02 (0.01; 0.03)
Plasma glucose^b^							
Crude	Ref	− 0.07 (− 0.12; − 0.02)	− 0.04 (− 0.09; 0.01)	− 0.10 (− 0.15; − 0.05)	− 0.06 (− 0.11; − 0.01)	0.005	− 0.02 (− 0.03; − 0.01)
Multiple adjusted 1	Ref	− 0.07 (− 0.12; − 0.02)	− 0.04 (− 0.09; 0.02)	− 0.09 (− 0.14; − 0.04)	− 0.06 (− 0.11; − 0.01)	0.015	− 0.02 (− 0.03; − 0.01)
Multiple adjusted 2	Ref	− 0.08 (− 0.13; − 0.03)	− 0.04 (− 0.09; 0.01)	− 0.11 (− 0.16; − 0.05)	− 0.07 (− 0.12; − 0.02)	0.002	− 0.02 (− 0.03; − 0.01)

*HDL-c* high-density lipoprotein-cholesterol

^a^MM-type estimators for linear robust regression models, the betas represent the change in each outcome, where 1 unit is equivalent to a 1-SD difference in z scores, or a 1-unit difference in the MetS z-score or its components, per one point of dietary adherence to PVG food patterns, either in the continuous (per each 5 points of adherence) or quintiles form of the different PVG food patterns

^b^Data were standardized

^c^*p* *trend* test for linear trend were conducted using the adherence to a uPVG food pattern quintile, *Crude* adjusted for energy intake *multiple adjusted 1* additionally adjusted for sex and age, *multiple adjusted 2* additionally adjusted for educational level, *smoking status* alcohol intake, and total physical activity per day

When we specifically assessed the hPVG version, the associations were stronger. For the fully adjusted models (Table 4), the hPVG was associated with lower MetS z-scores (β for Q5 vs Q1 = − 0.23; 95% CI: − 0.41 to − 0.05; *p* trend: 0.016), BMI (β for Q5 vs Q1 = − 0.07; 95% CI: − 0.15 to 0.02; *p* trend: 0.043) and WHR (β for Q5 vs Q1 = − 0.14; 95% CI: − 0.22 to − 0.07; *p* trend: < 0.001). We also observed inverse associations between the adherence to hPVG (per 5 points of increment) and MetS z-score, *β* = − 0.06 (95% CI: − 0.10; − 0.02) and WHR *β* = − 0.04 (95% CI: − 0.05; − 0.02).

By contrast, in the fully adjusted models for the uPVG (Table 5) we observed a significant positive association with the MetS z-score (β for Q5 vs Q1 = 0.21; 95% CI: 0.04 to 0.38; *p* trend: 0.019), DBP (β for Q5 vs Q1 = 0.08; 95% CI: 0.00 to 0.15; *p* trend: 0.042) and plasma triglycerides (β for Q5 vs Q1 = 0.08; 95% CI: 0.02 to 0.13; *p* trend: 0.003). In addition, we observed an inverse association with HDL-cholesterol (β for Q5 vs Q1 = − 0.11; 95% CI: − 0.18 to − 0.04; *p* trend: 0.001) and plasma glucose (β for Q5 vs Q1 = − 0.07; 95% CI: − 0.12 to − 0.02; *p* trend: 0.002). In the models with continuous variables, we also observed inverse associations per each 5 points increases in adherence to the uPVG of *β* = − 0.02 (95% CI: − 0.04; − 0.01) for HDL-cholesterol and *β* = − 0.02 (95% CI: − 0.03; − 0.01) plasma glucose, and direct associations of *β* = 0.02 (95% CI: 0.01; 0.03) for plasma triglycerides.

## Discussion

The results of this study suggest that adults with MetS and a higher adherence to the general and hPVG food patterns showed more favorable cardiometabolic markers as measured by the MetS z-score and several of its components. On the contrary, those participants with higher adherence to the uPVG food pattern showed worse cardiometabolic markers.

Although the research about these food patterns is relatively recent, several studies have shown consistent results. A prospective cohort study in South Korean investigated the role of being adherent to four plant-based diet indices (PDI) and found a positive linear association between higher adherence to an unhealthful plant-based diet (uPDI) and the incidence of MetS [28]. In the Adventist Health Study 2, a prospective study with 96,000 participants of the Seventh-day Adventist church mostly following vegetarian diets, positive associations were found between vegetarian diets and all components of metabolic syndrome (triglycerides, DBP, SBP, waist circumference, BMI and glucose), although not so for HDL-c [29]. Two other prospective cohort studies in the USA and Spain have also found beneficial associations for weight change in the case of the gPVG and hPVG food patterns [17, 30], in line with our findings for BMI.

The beneficial effect of a PVG food pattern might extend beyond the improvement in cardiometabolic markers. In a previous research of 12,168 middle-aged adults in South Korea (45–64 years of age at baseline), a higher adherence to a healthful PDI index and PVG patterns was associated to lower risk of cardiovascular morbidity and mortality, and lower all-cause mortality [31]. A lower all-cause mortality was also reported for those with a gPVG food pattern in an older population of the PREDIMED study [14].

Participants with better adherence to uPVG food pattern showed lower plasma glucose concentrations in our study. This type of inverse association between uPDI food pattern and risk of T2D has been also shown previously [15]. An explanation for this unexpected association could be some reverse causation, in the sense that those subjects with a T2D diagnosis were more aware of sugar content in different food groups and for this reason we observed lower prevalence of T2D among those better adhering a uPVG food pattern.

The mechanisms by which PVG food patterns could have cardiometabolic beneficial effects are multiple, likely related to the high content of plant-based foods with low glycemic index [32]. A higher intake of plant foods like fruits, vegetables, nuts, legumes or whole grains, leads to a higher intake of different bioactive compounds such as fiber which has been associated with greater satiation and consequently, a lower energy intake and body weight [33]. Moreover, the consumption of different types of fiber can modulate and improve glucose homeostasis by different mechanisms such as a delay of gastric emptying with consequent reduction in glucose absorption or via its fermentation in the colon, that produces short-chain fatty acids, which may reduce glucose formation in hepatocytes [34, 35]. Other components of plant foods such as polyphenols or stanols, can reduce the endogen pathways of lipids formation. As shown in a systematic review and meta-analysis of observational and intervention studies, plant-based diets have been consistently associated with lower blood lipid levels such as total cholesterol, c-LDL and c-HDL [36]. Nitric oxide is another substance that we produce when take a sunlight bath or with the ingestion of some nutrients present in plant foods, like the amino acid L-arginine present in seeds and nuts [37] or nitrates present in various vegetables including beets [38], which could improve blood pressure and endothelial and platelet function through different mechanism.

Conversely, uPVG may increase cardiometabolic risk because some of its components, such as chips, sugar-sweetened beverages, sugary desserts, sweets, are rich in added sugars, sodium, poor quality fats, refined starches and flavor enhancers. Many of these foods usually belong to ultra-processed food groups that could damage our internal systems, worsening our glucose homeostasis, increasing our blood pressure and modifying the ratio of blood lipids to a pattern of increased cardiometabolic risk, regardless of whether they come from plants or animals [39–41].

Apart from the potential beneficial effects of PVG food patterns, we should consider their environmental consequences. Thus, in one analysis performed in the SUN Project which compared this pattern with other options as MedDiet or Western Diet, despite the fact that MedDiet presents the relatively lowest environmental footprint, gPVG food pattern was the eco-friendliest pattern and with the additional advantage of being more affordable when compared to MedDiet [42].

We acknowledge that our study has several limitations. First, cross-sectional studies have a limited capacity to establish causality, and may be prone to reverse causation as that mentioned above for the inverse association between uPVG and T2D. However, there are previous studies, some of them with prospective design, that showed results in the same direction as our findings. Second, we took into account in the analyses several confounders such as sociodemographic or lifestyle variables, but there may be other potential confounders not accounted for that may influence cardiometabolic risk. Another limitation is our diet measurement instrument. Although it was a validated instrument, it refers to the usual intake over the previous year and therefore makes it difficult to draw conclusions about the longer-term effects of the diet on cardiometabolic risk. Another limitation of our study concerns the study population, elderly people with metabolic syndrome and without prior cardiovascular events, which make it difficult to extrapolate results to other healthy or dissimilar populations. Thus, it is desirable to replicate our results in future studies with different populations.

Our study has also strengths. The quality and quantity of information that we measured is high thanks to our trained personal and the robustness of our findings, that were maintained after adjusted the models for possible confounders. Additionally, the use of three plant-based dietary patterns with a better assignment of several specific foods helped us to distinguish that not all vegetarian patterns are as beneficial as supposed to be. Our findings may also help to clarify some inconsistencies in the literature and to determine which type of dietary recommendations may be most beneficial when following a PVG pattern to reduce the overall MetS risk.

In conclusion, this study suggests that among older adults at high cardiometabolic risk, a greater adherence to general and hPVG food patterns are associated with lower cardiometabolic risk, while a greater adherence to uPVG food pattern is associated with a higher cardiometabolic risk. Further studies are recommended to investigate if these associations are also observed in other healthy populations.

## Abbreviations

PVG: Pro-vegetarian
gPVG: General pro-vegetarian
hPVG: Healthful pro-vegetarian
uPVG: Unhealthful pro-vegetarian
SUN: Seguimiento Universidad de Navarra
MetS: Metabolic syndrome
MetS z-score: Metabolic syndrome z-score
CVD: Cardiovascular disease
DBP/SBP: Diastolic and systolic blood pressure
BMI: Body mass index
MedDiet: Mediterranean diet
T2D: Type 2 diabetes
FFQ: Food frequency questionnaire
WHR: Waist to hip ratio
EDTA: Ethylene diamine tetraacetic acid
HDL-c: High density lipoprotein cholesterol
METS: Metabolic equivalents
SD: Standard deviation
Q: Quintile
CI: Confidence interval
PDI: Plant-based diet index
uPDI: Unhealthful plant-based diet index
